# Ovarian Folliculogenesis and Uterine Endometrial Receptivity after Intermittent Vaginal Injection of Recombinant Human Follicle-Stimulating Hormone in Infertile Women Receiving In Vitro Fertilization and in Immature Female Rats

**DOI:** 10.3390/ijms221910769

**Published:** 2021-10-05

**Authors:** Chao-Chin Hsu, Leonard Hsu, Yuan-Shuo Hsueh, Chih-Ying Lin, Hui Hua Chang, Chao-Tien Hsu

**Affiliations:** 1 Taiwan United Birth-Promoting Experts Fertility Clinic, Tainan 710, Taiwan; tube2363808@gmail.com; 2 Department of Obstetrics and Gynecology, National Cheng Kung University Hospital, Tainan 701, Taiwan; 3 Department of Obstetrics and Gynecology, China Medical University, Taichung 404, Taiwan; 4 Department of Obstetrics and Gynecology, National Taiwan University Hospital, Taipei 100, Taiwan; 5 Douglas Mohr Pathology, Sydney, NSW 2113, Australia; leon26hsu@gmail.com; 6 Department of Medical Science Industries, College of Health Sciences, Chang Jung Christian University, Tainan 711, Taiwan; yshsueh@mail.cjcu.edu.tw; 7 Institute of Clinical Pharmacy and Pharmaceutical Sciences, College of Medicine, National Cheng Kung University, Tainan 701, Taiwan; ilg811280@gmail.com; 8 School of Pharmacy, College of Medicine, National Cheng Kung University, Tainan 701, Taiwan; 9 Department of Pharmacy, National Cheng Kung University Hospital, College of Medicine, National Cheng Kung University, Tainan 701, Taiwan; 10 Department of Pharmacy, National Cheng Kung University Hospital, Dou-Liou Branch, Yunlin 640, Taiwan; 11 Department of Pathology, E-Da Hospital, Kaohsiung 824, Taiwan; ed103797@edah.org.tw

**Keywords:** recombinant human follicle-stimulating hormone, gonadotropin, vaginal administration, uterine first-pass effect, ovarian folliculogenesis, endometrial receptivity, in vitro fertilization, pharmacokinetic, vaginal injection, progesterone receptor

## Abstract

The uterine first-pass effect occurs when drugs are delivered vaginally. However, the effect of vaginally administered recombinant human follicle-stimulating hormone (rhFSH) on ovarian folliculogenesis and endometrial receptivity is not well established. We aimed to compare the efficacy of rhFSH administered vaginally and abdominally in clinical in vitro fertilization (IVF) treatment, pharmacokinetic study, and animal study. In IVF treatment, the number of oocytes retrieved, endometrial thickness and uterine artery blood perfusion were not different between women who received the rhFSH either vaginally or abdominally. For serum pharmacokinetic parameters, significantly lower Tmax, clearance, and higher AUC and T_1/2__elimination of rhFSH were observed in women who received rhFSH vaginally, but urine parameters were not different. Immature female rats that received daily abdominal or vaginal injections (1 IU twice daily for 4 days) or intermittent vaginal injections (4 IU every other day for two doses) of rhFSH had more total follicles than the control group. In addition, the serum progesterone and progesterone receptors in the local endometrium were significantly higher in the groups treated with intermittent abdominal or vaginal injection of rhFSH, compared with those who recieved daily injection. In summary, vaginal administration of rhFSH may provide an alternative treatment regimen in women receiving IVF.

## 1. Introduction

The World Health Organization defined infertility as “a disease of the reproductive system by the failure to achieve a clinical pregnancy after 12 months or more of regular unprotected sexual intercourse” [1]. It is estimated to affect between 8 and 12% of reproductive-aged couples worldwide [2]. In vitro fertilization (IVF) is the most effective form of assisted reproductive technology. There are at least five major prerequisites necessary to achieve successful IVF pregnancies in humans which include obtaining oocytes that are sufficiently mature, obtaining suitable sperm cells which are acrosome-reacted, identifying suitable media for the culture of oocytes, spermatozoa and for the in vitro fertilization it-elf, ensuring the safe transfer of embryos to the uterus, and supporting the implantation and maintenance of embryos [3]. Among those, the detection and retrieval of mature oocytes from a human ovary is the first mandatory pre-requisite and controlled ovarian stimulation (COS) involving gonadotropin (Gn) administration to stimulate the growth of multiple ovarian follicles is the most effective way to increase pregnancy rates in in vitro fertilization (IVF) treatments [4,5]. COS depends on ovarian reserve, and there is individual heterogeneity in the ovarian response to exogenous Gn, especially the follicle-stimulating hormone (FSH) [6,7]. Previous systematic reviews and meta-analyses have indicated that an elevated number (>15) of oocytes retrieved was associated with higher numbers of good-quality embryos and frozen embryos, subsequently leading to increased cumulative pregnancy rates [8,9,10]. Women undergoing IVF treatment must receive COS to promote multiple follicle growth to achieve better outcomes. In the past decade, there has been a shift of practice from retrieving ‘the more oocytes, the better’ to retrieving an optimal range of oocytes that yields the maximal chance of conceiving [11,12]. A more patient-friendly approach with respect to the mode of administration of Gn, including FSH, has become a popular trend in personalized IVF treatment [13].

Based on the FSH threshold [14], a certain level of FSH should be exceeded to enhance FSH-dependent preantral and early antral follicle progression to maturation [15]. Pharmacokinetic studies showed that the terminal half-life (t_1/2_) of recombinant FSH was only 17 to 35 h for follitropin alpha and beta, respectively [16,17]. Thus, it is universally believed that daily administration of FSH to maintain an adequate serum FSH concentration is the single most important factor in COS [18]. However, daily injection of Gn is very stressful for most women [19], and accurate Gn injections that minimize pain, difficulty, and stress are essential for patient compliance [20]. Individualized versus standard FSH dosing in women with a predicted hyper response or poor ovarian reserve starting IVF have been extensively investigated in randomized controlled studies [21]. The application of GnRH-antagonist further reduced the risk of ovarian hyperstimulation syndrome (OHSS) in women with predicted hyper-response such as polycystic ovarian syndrome (PCOS) [22]. In the era of increasing demand for more efficient ovarian stimulation, as many advanced aged women and more women of poor ovarian reserve request IVF treatments, different administration modes of recombinant human FSH (rhFSH) might provide each patient an individualized tailored scheme to optimize the response of COS. Our recent studies proved that rhFSH can be administered intradermally and effectively absorbed and delivered to the ovaries to enhance the follicle growth [23,24]. The rhFSH in combination with platelet rich plasma has recently been directly injected into the target ovarian tissue and ovarian subcortical area to restore the ovarian function and achieve clinical pregnancy in women of premature ovarian insufficiency and early menopause [25,26].

**Figure 1:**
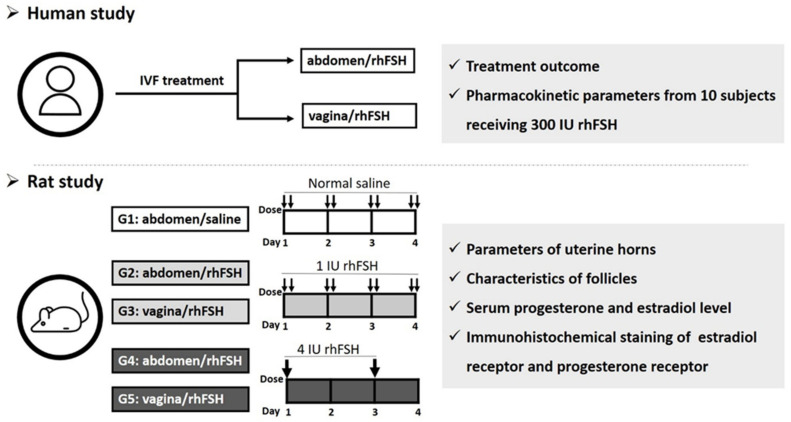
The overall scheme of study design. Abbreviations: rhFSH: recombinant human follicle-stimulating hormone.

A “uterine first-pass effect” that occurs when steroid hormones are delivered through the vagina has been suggested [27]. Vaginal administration of progesterone supplements in the luteal phase has been proven to have uterine effects that exceed the response that can be expected from the circulating levels of progesterone [28,29], with ~10-fold progesterone delivery to the uterus in ~7-fold lower circulating concentrations than intramuscular injections [30,31]. In our previous pilot study, we demonstrated that intermittent vaginal injection of rhFSH in women receiving IVF treatment resulted in proper folliculogenesis, fertilization rate, implantation rate, and pregnancy rates [32,33]. However, information about the effect of FSH on ovarian folliculogenesis and uterine endometrial receptivity after vaginal administration in female subjects receiving IVF has not been well established. Furthermore, information about the effects of vaginal administration on the morphology and functional parameters, including estrogen receptor (ER) and progesterone receptor (PR), of the uterine horn and on ovarian folliculogenesis patterns in animal models remains scarce. In the current study, we investigated the effect of rhFSH on ovarian follicle and uterine parameters after abdominal and vaginal administration in infertile women receiving IVF. In addition, we compared the pharmacokinetic parameters determined from serum and urine samples in 10 non-infertile women who received 300 IU rhFSH between abdominal and vaginal administrations. Furthermore, we investigated the effect of abdominally and vaginally injected rhFSH on the uterine endometrium, ovarian follicle growth, and functional parameters (ER and PR) in female rats. The overall scheme of the study design is shown in Figure 1. The results of this study suggest various scenarios for COS in clinical IVF treatment.

## 2. Results

### 2.1. Human Study

The clinical parameters of age and anti-Müllerian hormone (AMH)-matched subjects who received rhFSH either using conventional abdominal administration or intermittent vaginal administration of rhFSH were compared. The demographic characteristics of the subjects are shown in Table 1. Both groups of women had poor ovarian reserve (POR) with low serum AMH levels (0.80 ± 0.32 and 0.72 ± 0.37 pg/mL, respectively). The causative factors of infertility included ovulatory (55% and 53%), endometriosis and/or adenomyosis (20% and 19%), tubal/pelvis factor (12% and 14%), male factor (10% and 12%), and others. Thus, both groups of women were homogeneous in their baseline before treatment. Approximately two-thirds of rhFSH doses (2014.77 ± 489.16 vs. 2905.43 ± 967.40 IU, respectively) were required to complete the COS process under intermittent vaginal administration. No difference was noted in the growth of ovarian follicles or in the number of oocytes retrieved. A lower peak serum concentration of estradiol (*p* = 0.053) and slightly higher progesterone (*p* = 0.078) were noted in women who received intermittent vaginal administration on the day of ovulation triggered by human chorionic gonadotropin (hCG). The uterine endometrial thickness and bilateral uterine artery blood perfusion presented by the pulsatility index and the resistance index did not differ between the two groups of participants. Although the number of embryos transferred was slightly lower in women who received vaginal injection, the pregnancy rate did not differ between the two groups. No discomfort, bleeding or painful sensation was noted in the women who received vaginal administration of rhFSH, albeit almost 80% of women were unaware of injection after the completeness of the rhFSH vaginal administration. More satisfaction was shown by most women for the intermittent vaginal administration instead of daily injection.

The ovarian sensitivity indices (OSIs) of 755.3 ± 639.9 and 768.7 ± 532.6 IU observed in the present study indicated that women with POR might achieve proper follicle maturation under both methods of administration of rhFSH. We also observed a follicular output rate (FORT) level of 60% and a follicle to oocyte index (FOI) of around 85% in the present study, indicating the dynamic response of ovarian folliculogenesis to exogenous Gn and the efficacy of both administration modes.

To investigate whether the injection site of rhFSH influenced the pharmacokinetic parameters, serum and urine data were collected from 10 subjects who received 300 IU rhFSH through crossover abdominal and vaginal administration (Table 2). The serum FSH data showed that female subjects who received rhFSH through vaginal administration had faster absorption and slower elimination represented by a significantly lower time of maximum observed concentration (Tmax), CL and elimination rate constant (K_10_), a greater area under curve (AUC), and T_1/2__elimination, compared with those who received rhFSH through abdominal administration. In addition, injection at different sites did not alter the estradiol pharmacokinetic parameters determined from serum samples (Supplementary Table S1). However, the FSH pharmacokinetic parameters determined from urine samples, including mean residence time from beginning to last observation (MRT), AUC, cumulative elimination dose, percentage of cumulative elimination dose, and urine clearance of rhFSH, did not differ significantly between two modes of rhFSH administration. (Table 2).

### 2.2. Rat Study

The animals’ mean body weights, including ovarian, uterine, uterine/body, and ovarian/body weight did not differ significantly among the groups (Supplementary Table S2).

#### 2.2.1. Parameters of Uterine Horns

The folding of the endometrial layer, the diameter of the uterus, the total area of the uterus, and the area of the endometrial layer did not differ among the groups, whereas the height of the endometrial epithelial cell layer was significantly different (*p* = 0.017) (Table 3).

#### 2.2.2. Characteristics of Follicles

**Figure 2:**
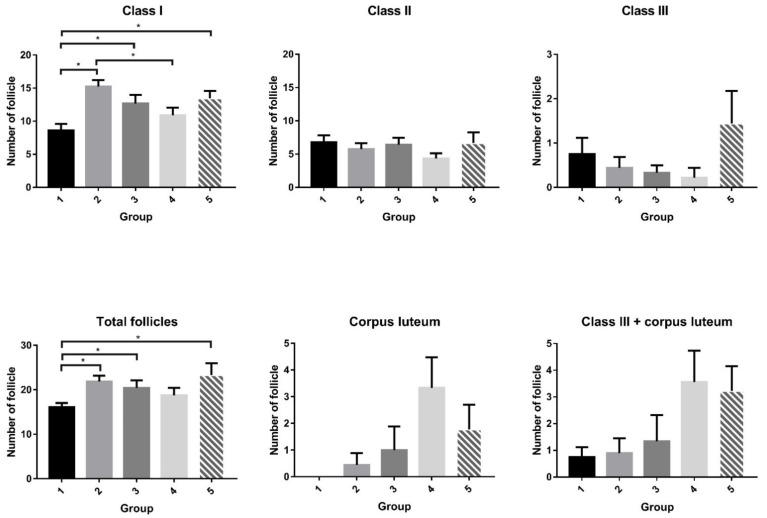
Number of follicles of each class in rats. In the ovary, the number of smaller follicles differed significantly among the groups (*p* = 0.007). There were more smaller follicles in most groups of rats that received FSH injections (groups G2, G3, and G5), regardless of injection frequency and site, than in the negative control (group G1). Although the ovarian follicle patterns of large- (class III) and medium- (class II) sized follicles showed no relationship to injection frequency or site, the total number of follicles differed significantly among the groups (*p* = 0.041). The rats that received abdominal (G2) or vaginal (G3) injections of 1 IU rhFSH or vaginal (G5) injection of 4 IU rhFSH had more follicles than the controls. In addition, the number of corpora lutea was borderline different among the groups (*p* = 0.064), and the rats that received abdominal injections of 4 IU rhFSH (G4) had significantly more corpora lutea than the control rats. Moreover, there was a trend toward an increasing number of large follicles and corpora lutea in rats that received abdominal (G4) or vaginal (G5) s.c. injections of 4 IU rhFSH. G1: abdominal s.c. injection of normal saline twice daily for 4 days (days 1 to 4); G2: abdominal s.c. injection of rhFSH (1 IU) twice daily for 4 days (days 1 to 4); G3: vaginal s.c. injection of rhFSH (1 IU) twice daily for 4 days (days 1 to 4); G4: abdominal s.c. injection of rhFSH (4 IU) every other day (2 injections) (days 1 & 3); G5: vaginal s.c. injection of rhFSH (4 IU) every other day (2 injections) (days 1 & 3). * *p* < 0.05.

In the ovary, there were more small follicles in the groups of rats that received rhFSH injections, regardless of injection frequency and site, than in the negative control (Table 3 and Figure 2). Over 90% of the observed ovarian follicles were atretic, and this phenomenon was widely expressed among small, medium, and large follicles. The rats that received abdominal (G2) or vaginal (G3) s.c. injections of rhFSH (1 IU), and those that received vaginal (G5) s.c. injections of rhFSH (4 IU), had more follicles than the controls. There was a trend of an increasing number of large follicles and corpora lutea in rats that received abdominal (G4) or vaginal (G5) s.c. injections of 4 IU rhFSH. There was no difference in the expression of cytochrome P450 aromatase activity in the ovarian tissue in any of the groups of rats analyzed (data not shown).

#### 2.2.3. Serum Progesterone Level and Detection of ER and PR

**Figure 3:**
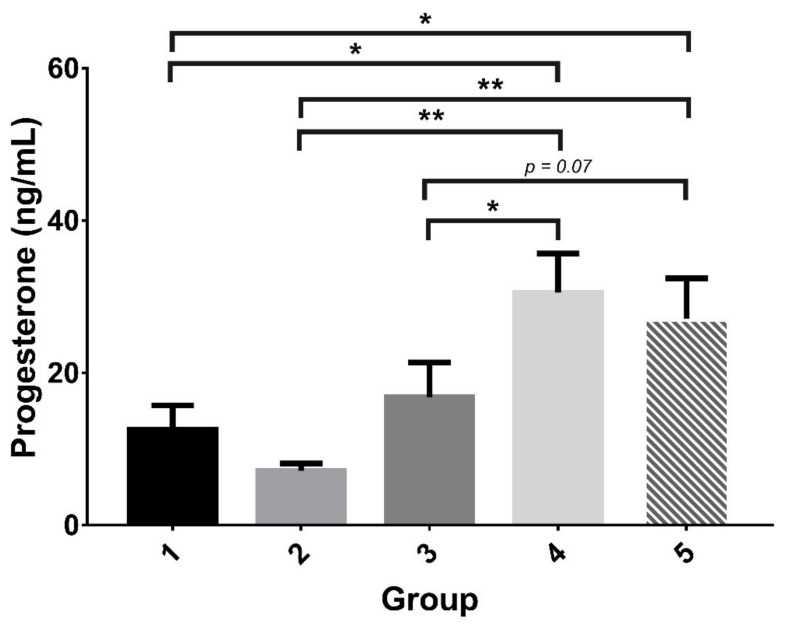
Serum progesterone levels in rats. The serum progesterone levels in the groups that received intermittent abdominal (G4) or vaginal (G5) s.c. injection of rhFSH were significantly higher than those in the groups that received daily abdominal (G2) or vaginal (G3) s.c. injection of rhFSH. The progesterone levels in the groups that received intermittent abdominal (G4) or vaginal (G5) s.c. injection of 4 IU rhFSH were significantly higher than that of the control (G1). G1: abdominal s.c. injection of normal saline twice daily for 4 days (days 1 to 4); G2: abdominal s.c. injection of rhFSH (1 IU) twice daily for 4 days (days 1 to 4); G3: vaginal s.c. injection of rhFSH (1 IU) twice daily for 4 days (days 1 to 4); G4: abdominal s.c. injection of rhFSH (4 IU) every other day (2 injections) (days 1 & 3); G5: vaginal s.c. injection of rhFSH (4 IU) every other day (two injections) (days 1 & 3). * *p* < 0.05, ** *p* < 0.01.

**Figure 4:**
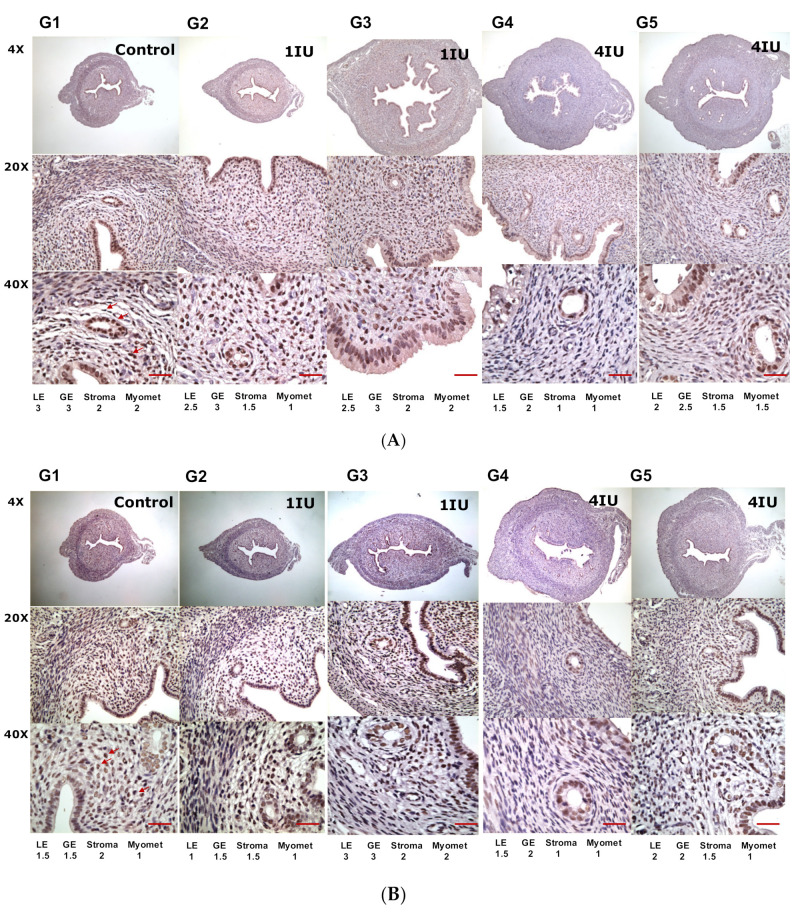
The expression of estradiol receptors and progesterone receptors in the uterus of rats after rhFSH treatment. For the detection of (**A**) estradiol receptor and (**B**) progesterone receptor by immunohistochemical staining. The degrees of staining were categorized as follows: (0), no staining; (1), faint staining; (2), moderate staining; and (3), strong staining. Arrows pinpointed the staining of ER or PR. Scale bar = 500 μm. G1: abdominal s.c. injection of normal saline twice daily for 4 days (days 1 to 4); G2: abdominal s.c. injection of rhFSH (1 IU) twice daily for 4 days (days 1 to 4); G3: vaginal s.c. injection of rhFSH (1 IU) twice daily for 4 days (days 1 to 4); G4: abdominal s.c. injection of rhFSH (4 IU) every other day (two injections) (days 1 & 3); G5: vaginal s.c. injection of rhFSH (4 IU) every other day (two injections) (days 1 & 3). Abbreviations: LE: luminal endometrium; GE: glandular endometrium; Myomet: myometrium.

The serum level of progesterone in rats that received abdominal (G4) or vaginal (G5) s.c. injection of 4 IU rhFSH was significantly higher than the levels in most other groups (Table 3 and Figure 3). Figure 4 showed the expression of ER and PR by immunohistochemical staining. There were trends of decreased ER expression and enhanced PR expression in the rats that received rhFSH (G2 to G5), regardless of frequency or injection site, compared to the control. Markedly suppressed expression of ER in the luminal epithelium and stroma were noted. The expression of PR in the uterine epithelium was, however, enhanced (groups G2 to G5), especially in the groups that received vaginal injections (G3 and G5), compared to the negative control group (G1).

## 3. Discussion

In the current study, we found that women who received rhFSH through vaginal administration had faster absorption, slower elimination, and a larger AUC of rhFSH in serum samples than women who received rhFSH through abdominal injection. In clinical IVF treatments, non-inferiority findings were noted compared to the conventional daily SC injection in which infertile women with POR who received rhFSH through intermittent vaginal administration had similar growth of follicles and numbers of mature oocytes retrieved. Furthermore, in the animal study, we found that less frequent injection of FSH, either abdominally or vaginally, appeared to be more efficient in inducing follicular growth and ovulation was manifested by an enhanced numbers of larger follicles and corpora lutea. The height of the epithelial cell layer of the endometrium was higher in rats that received FSH through intermittent vaginal injection. In addition, the serum levels of progesterone and the expression of PR in the local endometrium were significantly higher in animals that received intermittent abdominal or vaginal injections of rhFSH than in animals that received daily abdominal or vaginal injections. In summary, our findings show that vaginal administration of rhFSH may provide an alternative treatment regimen in women receiving IVF.

In human IVF, the live birth rate per inseminated oocyte is extremely low, on average approximately 2–4% [34]. Superovulatory treatments are thus employed in IVF to increase the yield of oocytes as the idea that more embryos result in higher pregnancy rates is widespread [34]. Our participants in IVF treatments belong to POR of POSEIDON group 4: patients older than 35 years old, with POR: AMH < 1.2 ng/mL, antral follicles at the beginning of menstrual cycle (AFC) <5 [35]. The clinical pregnancy rate of 24% in our participants is comparable to a randomized controlled trial of POR, in which the ongoing pregnancy rate was 12.8% for mild ovarian stimulation versus 13.6% for conventional ovarian stimulation [36], and a cumulative live birth rate of 12.92% in gonadotropin releasing hormone (GnRH) antagonist cycle [37]. The total dose of rhFSH used (2014.77 ± 489.16 IU) in this study, however, was lower than that (2905.43 ± 967.40 IU) of our patients who received conventional SC injections, and the suggested dosage of over 3000 IU rhFSH for conventional IVF treatment [38,39]. Our clinical data were also comparable to the study of Berkkanoglu et al. in which 3.7 to 4.6 mature oocytes were retrieved in POR women, while they used up to 3749 to 4575 IU rhFSH for COS [40]. Thus, the protocol in the present study could save nearly one-third up to 56% of the overall cost for rhFSH injection in conventional practice and greatly diminish the cost of each IVF cycle.

In the present study, faster absorption, slower elimination, and larger AUC of rhFSH provide a pharmacokinetic basis for vaginal administration of rhFSH. The Tmax of 5.31 ± 3.52 h, AUC_0–t_ 1723.57 h*mIU/mL, and extended half-life of 69.99 h indicated a much faster and extended absorption of rhFSH at the injection site compared to that of conventional SC injection. The results fit well with our previous pharmacokinetic study through vaginal administration of rhFSH in which Tmax of 6.67 h, the maximum observed concentration (Cmax) was 15.77 IU/L, and AUC was 1640 IU·h/L [41]. Both studies on vaginal administration showed smaller plasma elimination rate constant (0.011 and 0.01 h^−1^), and slower total body clearance (292.2 and 190 mL/h). The total body clearance of 0.19 L/h was slower than our work on abdominal SC injections of rhFSH of 0.40 L/h, and is slower than a previous study of 0.5–0.6 L/h in intravenous injection of 300 IU of rhFSH [16] and 0.75 L/h in SC injection of 150 IU of rhFSH [42]. These pharmacokinetic pictures of faster absorption and slower elimination employing vaginal administration may provide advantages over conventional injection of rhFSH.

In dynamic analysis of folliculogenesis, the OSI of 768.7 ± 532.6 IU observed in participants with AMH of 0.72 ± 0.37 indicated proper follicle maturation in POR women after intermittent vaginal administration of rhFSH [43]. Compared to the response of FOI around 50% for conventional COS [44], the FOI was 85% in the study group, indicating a much higher response in our subjects and supporting the increased efficacy of this new administration mode. In the present study, we also found no difference in the comparison of FORT and FOI between the study and control group. According to Alviggi et al. [44], FOI might better reflect the dynamic nature of follicular growth in response to COS, compared to the traditional markers of ovarian reserve. Thus, the present administration mode of rhFSH might provide a new scenario of COS for women with POR.

The follicle-stimulating hormone, mediated by cAMP-dependent signal transduction, enables granulosa cells (GC) to convert theca cell androgens into estradiol [27]. The cytochrome P450 aromatase and 17β-hydroxysteroid dehydrogenase type-1 (17β-HSD) in GC orchestrate the FSH-dependent conversion of androgens into estrone and estradiol [45]. In the present study, no increased expression of cytochrome P450 aromatase was well correlated with almost undetectable serum estradiol levels in all rats investigated. The poor superovulation in immature rats noted in the present study reflects the fact that the level of endogenous luteinizing hormone (LH) in these animals is inadequate to reach the required synergism of FSH/LH for folliculogenesis and ovulation [28].

Increased uterine weight in laboratory rodents has long been used as a bioindicator of the presence of estrogenic steroids [46,47]. Increased edema, hypertrophy, and hyperplasia of the endometrium represented the uterotrophic effects of estrogens [46]. In this study, no significant increase in gross parameters, including the diameter and weight of the uterine horns, was noted in any of the groups of rats analyzed. This finding also reflects the very low (undetectable) serum levels of estrogen in the animals used in the study. The rats received only a low dose of FSH (8 IU), and thus the levels of exogenous hormone may not have been high enough to induce the production of sufficient estrogen to cause an uterotrophic effect. Progesterone exerted its effect as an antagonist to the estrogenic uterotrophic effect, and thus a thinner endometrium might be expected in those groups. This pattern was also noted in the expression of PR in different portions (glandular epithelium, luminal epithelium, stroma, and myoma) of the uterine horn in the animals that received single injections of rhFSH. Downregulation of the expression of PR with higher secretion of progesterone during ovulatory status could also explain the pattern of lower PR in the groups that received intermittent injections of FSH.

Embryo implantation depends on development of the uterus to the receptive stage and attainment of implantation competency by the embryos. The coordinated effects of progesterone and estrogen are essential for these processes, which determine the window of implantation. The correct temporal–spatial elaboration and balance of various growth factors, cytokines, lipid mediators, transcription factors, and other molecules putatively regulated by steroid hormones is thought to play an important role in uterine preparation for implantation [48,49,50]. The actions of estrogen and progesterone are primarily mediated by their nuclear receptors, the estrogen receptors (ERs) ERα and Erβ, and the progesterone receptors (PRs) PR-A and PR-B, respectively [51]. The increase in progesterone levels during the early secretory phase is responsible for the expression of a myriad of proteins, many of which appear to be critical for normal implantation. In the current study, a lower peak serum concentration of estrogen (*p* = 0.053) and slightly higher progesterone (*p* = 0.078) were noted in women who received intermittent vaginal administration of rhFSH for COS on the day of ovulation triggered by hCG. We also found that intermittent vaginal administration of rhFSH significantly increased progesterone levels, and this effect may induce specific epithelial gene products directly by acting through PRs in epithelial cells or indirectly by stimulating stromal factors [51].

In mice, estrogen stimulates the proliferation and differentiation of luminal and glandular epithelia, whereas the proliferation and differentiation of the stroma require both progesterone and estrogen [52,53]. The diverse actions of progesterone include balancing the effects of estrogen and modulating maternal immunological surveillance toward the embryo [54]. PR-A null female mice are infertile due to severe abnormalities in ovarian and uterine function. PR-A knockout mice are sterile, primarily due to a complete lack of implantation and a marked reduction in ovulation [55]. PR action is potentiated by an immunophilin cochaperone, FK-506 binding protein 4 (FKBP52). Mice that lack PR are infertile due to the complete failure of ovulation, fertilization, and implantation, and female mice with targeted deletion of the Fkbp52 gene are infertile specifically because of implantation failure resulting from compromised uterine receptivity [56]. In the present study, reversed patterns of ER and PR were noted in almost all groups of rats. A previous study indicated that PR-A is the principal isoform responsible for the inhibition of ER action and that ligand-activated PR-A can inhibit the transcriptional activities of ER [57]. The effects of elevated PR levels in rats receiving intermittent rhFSH injections and the possible clinical application of this effect need further investigation.

Steroid action during early pregnancy is an important area of research because of the persistent low pregnancy success rates (~30%) in human IVF programs. One cause of reduced pregnancy rates is the transfer of IVF-derived embryos into uteri that are nonreceptive due to the high estrogen concentrations resulting from the ovarian stimulation [58,59]. Thus, concentrations of estrogen must be tightly controlled for successful implantation to occur [60]. In fact, there is evidence that ovarian stimulation leads to implantation failure and embryonic resorption in mice [61,62]. These results demonstrate the importance of maintaining a tight balanced action between estrogen and progesterone in the uterus. Lower peak serum concentrations of estradiol and slightly higher progesterone levels were noted in women who received intermittent vaginal injection compared to those women who received conventional abdominal injections of rhFSH. Our clinical applications of intermittent injections of gonadotropins employing doses lower than those used in conventional IVF programs yielded improved embryo implantation rates and higher pregnancy rates [23,33]. Future larger studies will be needed to clarify whether intermittent vaginal injection of rhFSH results in a well-balanced hormonal status for embryo implantation.

The lower genital tract, including the vagina and uterus, represent an ideal site for drug administration because it possesses advantages such as an opportunity to bypass first-pass metabolism, high permeability for low-molecular-weight drugs, considerable surface area for absorption, and a rich blood supply [63]. Our pharmacokinetic data showed enhanced local absorption of rhFSH injected through the vaginal route, reflecting the proficiency of this mechanism. However, the effectiveness of vaginal administration may depend on several intrinsic factors, including pH, temperature, enzymatic metabolism, clearance, and fluctuations in hormone levels during the menstrual cycle [64]. In addition, the mucosal layers of the female reproductive tract may constitute a broad repertoire of immune responses [65]. A study showed that genital epithelial cells express a wide range of pattern recognition receptors (PRRs) that promote their ability to recognize and differentially respond to various pathogens [65]. The PRRs found in the female reproductive tract include Toll-like receptors (TLRs) and NOD-like receptors, both of which play important roles in protecting the host against pathogenic invasion, in tissue adaptation and ultimately in successful reproduction, resulting in part from modulation of transcription factors and other genomic and epigenetic alterations in the endometrial epithelium [65,66]. Whether the administration of rhFSH through the vaginal mucosa might alter the microbial system and lead to changes in some potential molecular functions linked to cell metabolism, motility, genetic information, the immune system, and signaling processes [67] warrants future study.

Although the data in this study were obtained and analyzed carefully, this study has certain limitations, including a relatively small sample size. However, we have made comprehensive investigations both in humans and rats, and the results indicated the efficacy and non-inferiority of vaginal administration of rhFSH for COS. There has been great demand for assisted reproduction in the last two decades, especially due to the increased tendency of poor ovarian reserve and advanced aged-women requesting further child-bearing possibilities. Thus, larger prospective studies should be conducted to verify the efficacy and cost effectiveness of intermittent vaginal administration of rhFSH in clinical IVF treatment. This study did not include vaginal injection of normal saline as a control, since rats injected with normal abdominal saline were used as a negative control and with abdominal 1 IU rhFSH injection as a positive control, which is mimicked the current treatment in humans.

## 4. Materials and Methods

### 4.1. Human Study

The study was performed in accordance with the Declaration of Helsinki Good Clinical Practice and local regulatory requirements. All of the enrolled participants were informed of the benefits, risks, and potential adverse reactions associated with different modes of rhFSH administration. This study was approved by the Institutional Review Board for the Protection of Human Subjects (TSMH IRB/Protocol No. 18–115-B), and all study patients provided written consent. All patients included in the study were treated at the IVF Unit at the TUBE Fertility Clinic, Tainan, Taiwan, under a license from the Taiwan Department of Health Authority.

#### 4.1.1. Participants

##### Clinical IVF Treatment

A retrospective analysis was performed based on data from 90 women with POR who received IVF treatments during 2011 and 2013, 45 women received intermittent vaginal administration and another 45 age-matched women received conventional daily abdominal injections of rhFSH for COS. The inclusion criteria were infertile women of 25–45 years of age with a body mass index (BMI) of 18.0–26.0 kg/m^2^, and the presence of bilateral ovaries. The main exclusion criteria were women with a history of allergic reactions, disorders of coagulation (hemophilics, undergoing therapy with anticoagulants or antiplatelet agents), advanced endometriosis stage III–IV, history of recurrent miscarriage, unexplained infertility, use of hormonal preparations during the last 3 menstrual cycles, and women undergoing preimplantation genetic screening (PGS) or preimplantation genetic diagnosis (PGD). The antagonist COS protocol was used for both groups. The protocol for intermittent vaginal administration was in accordance with our previous publication [33]. In brief, Gonal-F (300 IU rhFSH in 0.5 mL) was aspirated into a 1-mL syringe attached with a 30-gauge needle. The rhFSH was injected subcutaneously into the vaginal wall every 3 days (cycle day 2, 5, 8, 11) until the maturation of follicles. The injection was performed with the needle angled at 15–30° towards the vaginal mucosa and injected in the bilateral upper portions of the vaginal wall at positions corresponding approximately to 3 o’clock and 9 o’clock. In conventional abdominal injections, women receive a daily SC injection of 150–225 IU rhFSH. GnRH antagonist (orgalutron 0.25 mg, NV Organon, Oss, The Netherlands) was started once the ovarian follicle reached 14 mm in size. The follow-up of ovarian follicular growth by ultrasound scanning was mostly arranged on day 5 and day 8, to day 11. Oocyte retrieval, fertilization, culture of fertilized embryos, and embryo transfer procedures were performed using routine procedures [25]. All patients received luteal phase support using micronized progesterone (600 mg/day) starting on the day of oocyte retrieval. Pregnancy was diagnosed by increasing serum concentrations of ß-HCG 12 days after embryo transfer and subsequent demonstration of the presence of an intrauterine gestational sac by ultrasonographic examination.

##### Pharmacokinetic Study

A randomized, two-period crossover study was conducted in ten women, each of whom underwent two trial cycles using alternative injection sites. The site for the first cycle of injections was randomized to be either the abdomen or the vagina. The women cooperated well with the requirements imposed by the study and had not taken oral contraceptive pills or other forms of hormone therapy for at least 3 months preceding the study. All volunteers were nonsmokers, ovulatory (with a mean cycle length of 26–32 days and intra-subject variation of ± 2 days), and lacked evidence of ovarian disease. The uterine cervix Pap smears for all volunteers were normal. Women with evidence of polycystic ovarian syndrome or ultrasound evidence of ovarian cysts were excluded from the study. All subjects were also devoid of any medical disorders such as hypertension, hepatic or renal disease, and endocrine abnormalities. The following pharmacokinetic analyses were also performed: time of maximum observed concentration (Tmax), maximum observed concentration (Cmax), area under curve (AUC), clearance (CL), volume of distribution (V), absorption rate constant (K_01_), half-life of absorption (T_1/2__absorption), elimination rate constant (K_10_), half-life of elimination (T_1/2__elimination), mean residence time from beginning to last observation (MRT last), and urine clearance (Cl_urine). All pharmacokinetic parameters were calculated by using first compartment methods as previously described.

##### Clinical Measurements

Follicular growth was detected by 2D ultrasound scanning (Aloka 900, Tokyo, Japan) performed by a single observer (C.C. Hsu) using a 5.0-megahertz transvaginal transducer. Follicle diameter was calculated as the mean diameter measured in two dimensions. The thickness of the uterine endometrium was measured at the time scheduled for the measurement of follicular growth on cycle day 2, day 8, and on the day of ovulation triggered by hCG, and bilateral uterine artery blood perfusion presented by the pulsatility index and the resistance index was detected employing color Doppler examination on the day of ovulation triggering by hCG. The dynamic aspects of the follicular response to COS were analyzed using the ovarian sensitivity index (OSI: the dose of rhFSH used divided by the number of mature oocytes obtained), the follicular output rate (FORT: the preovulatory follicle count (14–22 mm in diameter) on hCG day × 100 divided by the small antral follicle count (3–8 mm in diameter) at baseline), and the follicle-to-oocyte index (FOI: the number of oocytes obtained divided by the number of antral follicles at the beginning of COS). Oocyte retrieval, fertilization, culture of fertilized embryos, and embryo transfer procedures were performed using routine procedures [32]. Briefly, transvaginal oocyte retrieval was performed at approximately 36 h after the injection of hCG. Oocytes were fertilized in vitro or by intracytoplasmic sperm injection (ICSI) and were cultured to the 6–8 cell stage at day 3, or to the blastocyst stage at days 5–6 after oocyte retrieval. The day 3 cleavage stage embryos in 30 μL of culture medium were transferred to the uterine cavity using an Embryo Transfer ET Catheter (Sydney IVF, Bulb tip, Cook, Sydney, Australia) under guidance by transabdominal ultrasound (3.5 MHz, Aloka SSD 1700; Aloka, Tokyo, Japan). Pregnancy was diagnosed by elevated serum β-hCG at 12 days after embryo transfer, and clinical pregnancy was demonstrated by the appearance of an intrauterine gestational sac on ultrasonographic scanning 2 weeks later.

##### Safety Parameters

The injection sites were monitored for redness, itching, swelling, pain and bruising at 1, 24 and 72 h post injection. No abnormal discharge or lesions were reported or observed during pelvic examination or vaginal injection.

##### Measurement of Serum Hormone Levels

For the pharmacokinetic study in 10 volunteers, in addition to baseline samples, urine and peripheral blood samples were taken at the following time intervals after the injections: 0.5 (optional), 1, 2, 4, 6, 8, 10, 12, 14 (optional), 24, 48, 72, 96, and 120 h. Serum and urine samples were frozen at −20 °C until assessment. Serum samples were collected on day 2 at the early follicular phase and on hCG day for ovulation triggering from women who received clinical IVF treatment. The Beckman Coulter ACCESS immunoassay system was used in the hormone assay (UniCelDxl 800, Beckman Coulter, Brea, CA, USA) as previously described [41]. FSH and LH in serum were measured using a sequential two-step immunoenzymatic sandwich assay. AMH levels were measured in serum samples using a simultaneous 1-step immunoenzymatic (“sandwich”) assay. A competitive binding immunoenzymatic assay was used to measure estradiol and progesterone serum levels.

### 4.2. Animal Study

#### 4.2.1. Rats

All experiments were performed according to the National Institutes of Health Guidelines for Animal Research (Guide for the Care and Use of Laboratory Animals) and were approved by the Animal Experimentation Committee of China Medical University. Immature female Sprague-Dawley rats 3–4 weeks of age (65–90 g) were used. The animals were housed in a constant environment with a temperature of 21 °C and a light/darkness cycle of 14/10 h and fed standard pelleted food and tap water. The general care and housing of rats was conducted at the Animal Center at China Medical University.

#### 4.2.2. Experiments

To examine the role of rhFSH in follicular growth in rats, a previous model that employed twice daily injections of rhFSH for 4 days (a total dose of 8 IU rhFSH) was employed [68]. The rhFSH used was Gonal-F (each vial contains 5.5 micrograms of follitropin alfa equivalent to 75 IU rhFSH), which was dissolved in 7.5 mL of sterile solvent to make the injection volume of 0.1 mL equivalent to 1 IU rhFSH. The immature female rats in the experimental groups received rhFSH (8 IU) injections as described below. G1: abdominal s.c. injection of normal saline twice daily for 4 days (days 1 to 4); G2: abdominal s.c. injection of rhFSH (1 IU) twice daily for 4 days (days 1 to 4); G3: vaginal s.c. injection of rhFSH (1 IU) twice daily for 4 days (days 1 to 4); G4: abdominal s.c. injection of rhFSH (4 IU) every other day (two injections) (days 1 & 3); G5: vaginal s.c. injection of rhFSH (4 IU) every other day (two injections) (days 1 & 3).

After 4 days of treatment, 18 h after the last injection on day 4, diethyl ether anesthesia was administered, and the animals were exsanguinated by drawing blood from the abdominal aorta. Serum samples were frozen at −20 °C until assayed. The bilateral uterine horns and ovaries were removed and placed in culture medium, and the adhering tissue was removed to prepare them for subsequent assays. The weight and dimensions of each uterine horn and ovary were measured. One ovary was immediately frozen at −80 °C for further analysis. The other ovary was fixed in Bouin’s fluid, sectioned, and stained for histological examination of follicular development. The uterine horns were fixed in Bouin’s fluid, sectioned, and stained for microscopic analysis of diameter, epithelium thickness, and lumen parameters.

#### 4.2.3. Measurement of Endometrium and Classification of Follicles

For histological examination, the fixed ovaries and uterine materials were embedded in paraffin, and serial sections (6–10 µm) were prepared and stained with hematoxylin and eosin. The diameter, the height of the endothelial lining, the folding of the endometrium, the area of the endometrial layer, and the area of the uterine horn were also determined. For the diameter and height of the endometrium, two measurements through a perpendicular line across the uterine section were obtained, and the mean was used. Differential follicle counts were performed according to a previously described method [68,69]. The number of antral follicles and atretic follicles was determined in three sections per ovary. In one ovary of each animal, antral follicles were determined according to their diameters: class I (275–400 µm, small follicles), class II (401–575 µm, medium-sized follicles), and class III (>575 µm, large-sized follicles). The mean follicle diameter was calculated from the two perpendicular diameters in the section containing the oocyte nucleolus. The morphological characteristics of atretic follicles include degeneration and detachment of the granulosa cell layer from the basement membrane, the presence of pyknotic nuclei, and oocyte degeneration [70,71,72]. The presence of a corpus luteum was also determined.

#### 4.2.4. Hormone Analysis

Progesterone and estradiol were measured using commercially available kits (Diagnostic Systems Laboratories, Inc., Webster, TX, USA). Progesterone and estradiol concentrations in serum were measured using a competitive radioimmunoassay. The sensitivity of the assays was 2.2 pg/mL and 0.10 ng/mL, and the inter-assay coefficients of variation were 8.0% and 2.4% for estradiol and progesterone, respectively.

#### 4.2.5. Immunohistochemistry of ER and PR

Sections of tissue blocks were cut onto adhesive-coated glass slides (Instrumedics, Hackensack) at a thickness of 3 μm. The slides were dewaxed in xylene, rehydrated through a graded alcohol series, pressure-cooked in 10 mM citrate buffer (pH 6) for 7 min, and washed using TBS with 0.1% Tween 80 for 5 min. Endogenous peroxidase activity was blocked by 3% H_2_O_2_ treatment. After washing, the slides were incubated with primary antibodies against ER (MA5-14501, Thermo Fisher Scientific, Waltham, MA, USA) and PR (MA5-14505, Thermo Fisher Scientific) at RT for 1 h. Primary antibodies were detected following the user’s manual of the ChemMate DAKO EnVision kit (DAKO). The slides were incubated with the secondary antibody for 30 min and developed with 3,3-diaminobenzidine for 5 min. Slides were then counterstained with hematoxylin. The slides were read visually, and the immunoexpression levels of ER and PR were independently scored by two expert pathologists (Leonard Hsu and Chao Tien Hsu) according to the four-point system (score 0–3).

### 4.3. Statistical Analysis

Statistical analysis was performed using the Statistical Package for Social Sciences 23.0 (SPSS Inc., Chicago, IL, USA) and GraphPad Prism version 6.0 (GraphPad Software Inc., La Jolla, CA, USA). In the human study, categorical variables are expressed as numbers and percentages, while continuous variables are expressed as the means ± standard deviations (SD) unless otherwise specified. The results obtained in the rat study are presented as the mean ± standard error of the mean (SEM). Categorical variables were assessed using chi-square tests, while continuous variables were assessed using t-tests or paired t-tests. Statistical analyses of the data obtained in the rat study were performed by Kruskal-Wallis one-way ANOVA followed by Dunn’s post hoc tests. The level of significance was set at 0.05 for two-sided tests.

## 5. Conclusions

In summary, the results of our study indicate that the overall outcomes of subjects who received rhFSH through intermittent vaginal administration were not inferior to those of subjects who received rhFSH via the daily conventional abdominal administration. In addition, female subjects who received rhFSH through vaginal administration had faster absorption, slower elimination, and a larger AUC of rhFSH in serum samples, but the site of injection of FSH did not alter the urine pharmacokinetic parameters in this human study. Moreover, the data obtained in our animal study suggests that less frequent injection of rhFSH, either abdominally or vaginally, was more efficient in inducing follicular growth and ovulation, as manifested by enhanced numbers of larger follicles and corpora lutea. Therefore, vaginal administration of gonadotropins may provide an alternative treatment regimen in women receiving IVF treatment.

## Figures and Tables

**Table 1:** Demographic characteristics, serum hormone levels, and follicle counts in subjects who received rhFSH abdominally or vaginally.

	Abdominal Administration Conventional SC (*n* = 45)	Vaginal Administration (*n* = 45)	t	*p* Value
Characteristics	Mean ± SD	Mean ± SD		
Age (years)	38.24 ± 3.37	39.09 ± 3.94	1.092	0.278
BMI (kg/m^2^)	23.58 ± 3.30	22.55 ± 3.54	−1.435	0.155
Serum hormone levels and ovarian follicle counts at baseline (Day 2)				
AMH (ng/mL)	0.80 ± 0.32	0.72 ± 0.37	−1.087	0.280
FSH (IU/L)	7.97 ± 6.59	10.20 ± 7.76	1.414	0.161
Estradiol (pg/mL)	19.88 ± 17.01	16.53 ± 8.83	−1.051	0.297
Luteinizing hormone (IU/L)	1.48 ± 1.34	1.47 ± 1.50	−0.027	0.978
Progesterone (ng/mL)	0.66 ± 0.43	0.66 ± 0.26	−0.049	0.961
AFC_baseline (number)	4.86 ± 2.50	3.77 ± 1.91	−1.900	0.062
Total gonadotropin dose (IU)	2905.43 ± 967.40	2014.77 ± 489.16	−5.473	<0.001 *
Serum hormone levels and ovarian follicle counts on day of ovulation triggering by hCG				
Estradiol (pg/mL)	543.87 ± 365.60	390.44 ± 363.25	−1.963	0.053
Luteinizing hormone (IU/L)	2.61 ± 5.77	2.49 ± 4.32	−0.100	0.921
Progesterone (ng/mL)	1.07 ± 0.47	1.65 ± 1.85	1.807	0.078
Ovarian follicle count				
Follicles < 12 mm (number)	0.93 ± 0.96	1.00 ± 1.04	0.315	0.754
Follicles ≥ 12 and <16 mm (number)	1.56 ± 1.50	1.78 ± 1.65	−0.049	0.961
Follicles ≥ 16 mm (number)	2.83 ± 2.51	2.24 ± 3.45	0.668	0.506
Follicles < 12 mm/total follicles (%)	19.76 ± 18.87	22.52 ± 22.05	0.633	0.528
Follicles ≥ 12 and <16 mm/total follicles (%)	29.17 ± 22.34	37.61 ± 24.78	1.172	0.243
Follicles ≥ 16 mm/total follicles (%)	48.87 ± 23.01	43.27 ± 22.95	−1.225	0.107
Oocytes retrieved				
Total oocytes (number)	5.33 ± 3.39	4.67 ± 4.48	−1.051	0.297
Mature oocytes (number)	4.59 ± 3.29	3.70 ± 4.33	−1.435	0.160
Mature oocytes/total oocytes (%)	83.28 ± 17.09	77.54 ± 24.49	−1.265	0.210
OSI (IU)	755.3 ± 639.9	768.7 ± 532.6	−0.108	0.6541
FORT (%)	58.39 ± 26.62	60.8 ± 26.17	−0.431	0.5687
FOI (%)	82.86 ± 32.73	86.41 ± 27.13	−0.561	0.1613
Endometrial thickness (mm)	8.95 ± 1.97	8.78 ± 2.00	−0.385	0.701
UABF _R-PI	1.99 ± 0.80	1.97 ± 0.68	−0.109	0.913
UABF _R-RI	0.79 ± 0.18	0.83 ± 0.10	1.104	0.274
UABF _L-PI	2.25 ± 0.94	2.21 ± 0.82	−0.181	0.857
UABF _L-RI	0.85 ± 0.12	0.89 ± 0.19	0.931	0.355
Fresh ET_number	1.43 ± 0.63	1.48 ± 0.55	−0.411	0.507
Pregnancy n, (%;/case had ET)	7 (24.1%; 7/29)	10 (24.4%; 10/41)	-	0.909

Abbreviations: SC: subcutaneous; BMI: body mass index; AMH: anti-Müllerian hormone; FSH: follicle-stimulating hormone; AFC: antral follicles at the beginning of menstrual cycle, indicated by the presence of 3–8 mm small follicles at baseline; OSI: ovarian sensitivity index, the dose of rhFSH used divided by the number of mature oocytes obtained; FORT: follicular output rate, the ratio of preovulatory follicle count (14–22 mm in diameter) on hCG day × 100/small antral follicle count (3–8 mm in diameter) at baseline; FOI: follicle to oocyte index, the ratio between the number of oocytes obtained and the number of antral follicles at the beginning of COS; UABF: uterine artery blood perfusion detected by color Doppler ultrasound scanning; R: right side; L: left side; PI: pulsatility index; RI: resistance index; ET: embryo transfer. * *p* < 0.05.

**Table 2:** Demographic characteristics and pharmacokinetic parameters of FSH in subjects receiving 300 IU rhFSH through abdominal/vaginal administration.

	Abdominal Administration (*n* = 10)	Vaginal Administration (*n* = 10)	t	*p* Value
Characteristics	Mean ± SD	Mean ± SD		
Age (years)	31.2 ± 7.3			-
BMI (kg/m^2^)	19.4 ± 2.1			-
Pharmacokinetic parameters of FSH determined from serum samples				
Tmax (h)	14.97 ± 8.65	5.31 ± 3.52	−3.106	0.013 *
Cmax (IU/L)	13.02 ± 1.56	15.77 ± 4.33	1.827	0.101
AUC (IU×h/L)	1104.85 ± 227.16	1723.57 ± 595.46	3.171	0.011 *
CL/F (L/h)	0.28 ± 0.04	0.19 ± 0.06	−4.208	0.002 *
V/F (L)	16.89 ± 4.80	18.59 ± 3.96	1.184	0.267
K_01_ (h^−1^)	0.64 ± 1.55	1.75 ± 2.27	1.174	0.271
T_1/2__absorption (h)	8.06 ± 9.60	1.42 ± 1.11	−2.072	0.068
K_10_ (h^−1^)	0.02 ± 0.00	0.01 ± 0.00	−6.843	<0.001 *
T_1/2__elimination (h)	41.26 ± 8.10	69.99 ± 13.67	6.416	<0.001 *
Pharmacokinetic parameters of FSH determined from urine samples				
MRT last_urine (h)	132.00 ±101.98	124.80 ± 86.01	−0.270	0.787
AUC_urine (IU × h/L)	1160.34 ± 841.76	1072.64 ± 861.38	−0.151	0.880
Cumulative elimination dose_urine (IU)	46.74 ± 32.48	38.83 ± 30.94	−0.756	0.450
Percentage of cumulative elimination dose_urine (Cumulative elimination dose/300 IU × 100%)	15.58 ± 10.83	12.94 ± 10.31	−0.756	0.450
Cl_urine(L/h)	0.05 ± 0.04	0.05 ± 0.03	−0.454	0.650

Abbreviations: BMI: body mass index; FSH: follicle-stimulating hormone; Tmax: time of maximum observed concentration; Cmax: maximum observed concentration; AUC: area under curve; CL/F: clearance/bioavailability; V/F: volume of distribution/bioavailability; K_01_: absorption rate constant; T_1/2_: absorption: half-life of absorption; K_10_: elimination rate constant; T_1/2_: elimination: half-life of elimination; MRT last: mean residence time from beginning to last observation; Cl_urine: urine clearance. * *p* < 0.05.

**Table 3:** Measurement of parameters of uterine horns, number of follicles classified, and hormone analysis in the rat study.

Group	G1	G2	G3	G4	G5	Statistical Analysis	
Site		abdominal	vaginal	abdominal	vaginal		
Unit	0 IU (*n* = 8)	1 IU (*n* = 9)	1 IU (*n* = 9)	4 IU (*n* = 9)	4 IU (*n* = 9)		
	Mean ± S.E.M	Mean ± S.E.M	Mean ± S.E.M	Mean ± S.E.M	Mean ± S.E.M	*p* value	post hoc
Parameters of uterine horns							
Diameter of the uterus (μm)	1314.71 ± 82.18	1344.13 ± 132.88	1520.88 ± 85.19	1577.00 ± 93.89	1618.89 ± 113.61	0.162	-
Folding of the endometrial layer(μm)	2822.00 ± 190.39	3654.13 ± 775.06	4295.75 ± 724.88	3591.22 ± 559.06	3812.11 ± 701.09	0.769	-
Height of epithelial cell layer of the endometrium(μm)	12.77 ± 0.52	20.60 ± 3.12	19.30 ± 2.36	20.27 ± 2.07	23.76 ± 3.26	0.017 *	G1 < G2, G1 < G3, G1 < G4, G1 < G5
Total area of the uterus (μm^2^)	1258,524.14 ± 133,695.87	1298,781.75 ± 248,034.88	1599,036.38 ± 222,079.93	1660,589.89 ± 185,274.63	1880,074.33 ± 234,992.75	0.274	-
Area of the endometrial layer (μm^2^)	552,151.43 ± 53,531.43	561,074.75 ± 77,514.61	706,550.50 ± 69,589.57	611,389.89 ± 73,587.71	794,114.11 ± 119,099.95	0.268	-
Number of follicles of each class							
Class I	8.6 ± 0.9	15.2 ± 0.9	12.7 ± 1.3	10.9 ± 1.2	13.4 ± 1.1	0.007 *	G1 < G2, G1 < G3, G1 < G5, G4 < G2
Class II	6.7 ± 1.0	5.8 ± 0.9	6.4 ± 1.0	4.3 ± 0.8	6.7 ± 1.6	0.493	-
Class III	0.7 ± 0.3	0.4 ± 0.2	0.3 ± 0.1	0.2 ± 0.2	1.4 ± 0.7	0.472	-
Total number of follicles	16.1 ± 0.9	21.9 ± 1.3	20.4 ± 1.7	18.8 ± 1.7	23.3 ± 2.7	0.041 *	G1 < G2, G1 < G3, G1 < G5,
Corpus luteum	0.0 ± 0.0	0.4 ± 0.4	1.0 ± 0.8	3.3 ± 1.1	1.8 ± 0.9	0.064	G1 < G4
Class III + corpus luteum	0.8 ± 0.3	0.9 ± 0.6	1.3 ± 0.9	3.6 ± 1.2	3.2 ± 0.9	0.220	-
Hormone analysis							
Progesterone (ng/mL)	9.50 ± 1.38	6.35 ± 0.64	13.30 ± 3.42	30.54 ± 5.14	27.09 ± 5.33	0.004 *	G1 < G4, G1 < G5, G2 < G4, G2 < G5, G3 < G4
Estradiol (pg/mL)	0.1 ± 0.0	0.1 ± 0.0	0.1 ± 0.0	0.1 ± 0.0	0.1 ± 0.0	0.999	-

G1: abdominal s.c. injection of normal saline twice daily for 4 days (days 1 to 4) (negative control group); G2: abdominal s.c. injection of rhFSH 1 IU twice daily for 4 days (days 1 to 4) (positive control group); G3: vaginal s.c. injection of 1 IU rhFSH twice daily for 4 days (days 1 to 4); G4: abdominal s.c. injection of 4 IU rhFSH every other day (2 injections) (days 1 & 3); G5: vaginal s.c. injection of 4 IU rhFSH every other day (2 injections) (days 1 & 3). Class I, II, III follicles are defined as follows: class I (275–400 µm, small follicles), class II (401–575 µm, medium-sized follicles), and class III (>575 µm, large-sized follicles). Statistical analysis was performed by Kruskal-Wallis one-way ANOVA followed by Dunn’s post hoc tests. The *p* values were presented the results of Kruskal-Wallis one-way ANOVA among 5 groups. The post hoc tests indicated the mean difference between specific pairs of groups were statistically significant using Dunn’s post hoc tests. * *p* < 0.05.

## Data Availability

The data that support the findings of this study are available from Chao-Chin Hsu. Restrictions apply to the availability of these data, which were used under license for this study. The data presented in this study are available on request from Chao-Chin Hsu with the permission of Taiwan United Birth-promoting Experts Fertility Clinic.

## Supplementary Materials

The following are available online at https://www.mdpi.com/article/10.3390/ijms221910769/s1.
