# Supplementary material for: Ovarian Folliculogenesis and Uterine Endometrial Receptivity after Intermittent Vaginal Injection of Recombinant Human Follicle-Stimulating Hormone in Infertile Women Receiving In Vitro Fertilization and in Immature Female Rats
Source: Int J Mol Sci. 2021 Oct 5;22(19):10769. doi: 10.3390/ijms221910769 (PMC8509306; doi:10.3390/ijms221910769)
Supplement: Supplementary file 1 [file ijms-22-10769-s001.zip › ijms-1386615-supplementary.pdf]

Supplementary Table S1. Pharmacokinetic parameters of estradiol in the subjects receiving 300IU rhFSH through abdominal or vaginal administration.

Characteristics	Abdominal administration (n = 10) mean ± SD	Vaginal administration (n = 10) mean ± SD	t	p value
Pharmacokinetic parameters of E2 determined from serum sample				
Tmax (hr)	31.29 ± 9.45	35.78 ± 18.05	0.617	0.558
Cmax (IU/L)	178.35 ± 178.78	88.83 ± 41.77	-1.482	0.177
AUC (IU×hr/L)	16389.62 ± 20462.02	9653.75 ± 6700.9	-0.875	0.409
CL/ F (L/hr)	0.04 ± 0.03	0.05 ± 0.03	0.219	0.838
V/F (L)	1.33 ± 0.98	7.28 ± 8.39	2.331	0.058
K_{01} (hr^{-1})	0.04 ± 0.00	0.04 ± 0.02	0.503	0.634
$T_{1/2}$_absorption (hr)	20.49 ± 8.23	20.65 ± 10.63	0.041	0.973
K_{10} (hr^{-1})	0.03 ± 0.00	0.03 ± 0.01	-0.492	0.639
$T_{1/2}$_elimination (hr)	23.81 ± 8.24	41.4 ± 47.93	1.051	0.325

Abbreviations: Tmax: time of maximum observed concentration; Cmax: maximum observed concentration; AUC: area under curve; CL/F: clearance/ bioavalibility; V/F: volume of distribution/ bioavalibility; K_{01}: absorption rate constant; $T_{1/2}$_absorption: half life of absorption; K_{10}: elimination rate constant; $T_{1/2}$_elimination: half life of elimination. * $p < 0.05$.

Supplementary Table S2. The measurements of body weight and ovarian size in rat study.

Group	G1	G2	G3	G4	G5	Statistical analysis
site		abdominal	vaginal	abdominal	vaginal	
unit	0 IU	1 IU	1 IU	4 IU	4 IU	
	mean ± S.E.M	mean ± S.E.M	mean ± S.E.M	mean ± S.E.M	mean ± S.E.M	p value
Body weight (g)	91.25 ± 2.50	94.22 ± 1.15	94.67 ± 2.03	99.89 ± 3.35	94.67 ± 3.12	0.144
Right ovarian weight (g)	0.02 ± 0.00	0.03 ± 0.00	0.03 ± 0.00	0.03 ± 0.00	0.03 ± 0.00	0.109
Left ovarian weight(g)	0.02 ± 0.00	0.03 ± 0.00	0.03 ± 0.00	0.03 ± 0.00	0.03 ± 0.00	0.101
Uterus (g)	0.10 ± 0.02	0.12 ± 0.03	0.14 ± 0.03	0.15 ± 0.02	0.13 ± 0.00	0.182
Mean ovarian weight/ body weight (%)	0.02 ± 0.00	0.03 ± 0.00	0.03 ± 0.00	0.03 ± 0.00	0.03 ± 0.00	0.182
Uterus/body weight (%)	0.11 ± 0.02	0.12 ± 0.03	0.14 ± 0.03	0.15 ± 0.02	0.14 ± 0.02	0.626

The total dose of rhFSH was the same 8 IU among G2 to G5 for 4 days in rats. G1: abdominal s.c. injection of normal saline twice a day for 4 days (day 1 to 4); G2: abdominal s.c. injection of rhFSH 1 IU twice a day for 4 days (day 1 to 4); G3: vaginal s.c. injection of rhFSH 1 IU twice a day for 4 days (day 1 to 4); G4: abdominal s.c. injection of rhFSH 4 IU every other day for 2 injections (day 1 & 3); G5: vaginal s.c. injection of rhFSH 4 IU every other day for 2 injections (day 1 & 3). *$p < 0.05$.

Supplementary Table S3. The estimate of immunohistochemistry of estradiol receptor and progesterone receptor in rat.

Group	G1	G2	G3	G4	G5	Statistical analysis
site		abdominal	vaginal	abdominal	vaginal	
unit	0 IU	1 IU	1 IU	4 IU	4 IU	
	mean ± S.E.M	mean ± S.E.M	mean ± S.E.M	mean ± S.E.M	mean ± S.E.M	*p* value
Estrogen receptor (ER)						
luminal endometrium	3.00 ± 0.00	2.50 ± 0.00	2.50 ± 0.00	1.33 ± 0.44	1.83 ± 0.17	0.010*
Glandular endometrium	3.00 ± 0.00	3.00 ± 0.00	2.83 ± 0.17	2.33 ± 0.33	2.50 ± 0.00	0.083
Stroma	2.00 ± 0.00	1.50 ± 0.00	2.00 ± 0.00	1.17 ± 0.17	1.33 ± 0.17	0.014*
Myometrium	2.00 ± 0.00	1.50 ± 0.29	1.67 ± 0.33	0.83 ± 0.17	1.50 ± 0.29	0.114
Progesterone receptor (PR)						
luminal endometrium	1.25 ± 0.14	1.00 ± 0.00	2.25 ± 0.25	1.50 ± 0.20	2.00 ± 0.00	0.015*
Glandular endometrium	1.50 ± 0.20	1.25 ± 0.25	2.25 ± 0.25	2.00 ± 0.00	2.33 ± 0.17	0.022*
Stroma	1.50 ± 0.20	1.25 ± 0.25	2.00 ± 0.00	1.13 ± 0.13	1.83 ± 0.17	0.028*
Myometrium	0.88 ± 0.13	0.75 ± 0.25	1.25 ± 0.25	1.38 ± 0.24	1.33 ± 0.33	0.242

For the detection of (A) estradiol receptor and (B) progesterone receptor, the degrees of staining were categorized as follows: (0), no staining; (1), faint staining; (2), moderate staining; and (3), strong staining. There were trends of decreased ER expression and enhanced PR expression in the rats that received rhFSH (G2 to G5), regardless of frequency or injection site, compared to the control. Markedly suppressed expression of ER in the luminal epithelium and stroma was noted. The expression of PR in the uterine epithelium was, however, enhanced (G2 to G5), especially in the groups that received vaginal injections (G3 and G5), compared to the negative control group (G1). G1: abdominal s.c. injection of normal saline twice daily for 4 days (days 1 to 4); G2: abdominal s.c. injection of rhFSH (1 IU) twice daily for 4 days (days 1 to 4); G3: vaginal s.c. injection of rhFSH (1 IU) twice daily for 4 days (days 1 to 4); G4: abdominal s.c. injection of rhFSH (4 IU) every other day (2 injections) (days 1 & 3); G5: vaginal s.c. injection of rhFSH (4 IU) every other day (2 injections) (days 1 & 3).
